# *Segniliparus rugosus*–associated Bronchiolitis in California Sea Lion

**DOI:** 10.3201/eid1702.101511

**Published:** 2011-02

**Authors:** Richard H. Evans

**Affiliations:** Author affiliation: Pacific Marine Mammal Center, Laguna Beach, California, USA

**Keywords:** Segniliparus rugosus, California sea lion, zoonoses, bacteria, California, letter

**To the Editor:** Until now, *Segniliparus rugosus* has not been isolated from nonhuman animals or the environment (*1*). On April 14, 2010, a rescue team from Pacific Marine Mammal Center impounded an emaciated and unresponsive subadult female California sea lion (*Zalophus californicus*) stranded on the beach at San Onofre, California, USA. Physical examination showed the animal to be obtunded and emaciated (third-stage malnutrition), with moderate bradycardia, hypoventilation, and hypothermia. Euthanasia was elected because of a poor prognosis. Immediately before euthanasia, a blood sample was taken for a complete blood count and serum chemistry evaluation. A postmortem examination was conducted immediately after euthanasia.

The postmortem examination showed marked subcutaneous and visceral adipose tissue depletion, as well as moderate skeletal muscle loss, especially in the axial skeleton. In the lungs, a frothy, greenish, mucoid material exuded from several dozen bronchioles. Samples of the exudate were submitted for cytologic examination and bacterial culturing (IDEXX Laboratories, Irvine, CA, USA). Selected tissues were sampled and fixed in 10% neutral buffered formalin for histopathologic examination.

Complete blood count and serum chemistry analysis showed moderate anemia; relative neutrophilia and monocytosis; mild to moderate relative lymphopenia; moderate to markedly reduced albumin, globulin, and total protein levels; and elevated creatine kinase and alkaline phosphatase levels. Such values are common in California sea lions with severe malnutrition (starvation).

Cytologic examination of the bronchiolar exudate indicated large amounts of mucin with erythrocytes; occasional epithelial cells; and small to moderate numbers of eosinophils, neutrophils, monocytes, and lymphoid cells, characteristic of a mild to moderate, subacute, mixed bronchiolitis. Histologic examinations of 3 sections of lung showed 33 bronchioalveolar foci containing varying numbers of adult *Parafilaroides decorus* nematodes, without associated inflammation. Eleven other foci showed moderate to marked chronic inflammation, with nematodes in only 2 foci. Gram stain did not show bacteria in any of these foci. Lesions were not found in sections of liver, kidney, bladder, spleen, and heart.

A commercial veterinary laboratory (IDEXX Laboratories) isolated an acid-fast organism from the lung swab. This organism was referred to National Jewish Medical and Research Center (Denver, CO, USA) for species identification and sensitivity analysis. By 16S rDNA sequencing, the organism was identified as *S. rugosus*. Sensitivity testing showed that it was susceptible to rifabutin, cycloserine, clofazimine, moxifloxacin, ciprofloxacin, and clarithromycin and resistant to rifampin, streptomycin, amikacin, kanamycin, capreomycin, ethambutol, and ethionamide.

As in humans, this isolation of *S. rugosus* was associated with pathologic changes in the respiratory tract. Whether the relationship was causal or simply a fortuitous isolation of a previously unrecognized part of the normal respiratory flora is uncertain. However, a recent report by Sikorski et al. stated that *“*Environmental screens and metagenomic surveys did not detect a single phylotype… of the members of the genus *Segniliparus*” (*2*). In contrast, this case report begs the question of whether *S. rugosus* could be free-living in the oceans or part of the flora of any number of ocean-dwelling vertebrates or invertebrates.
